# Gleanings

**Published:** 1896-08

**Authors:** 


### GLEANINGS.

The Bureau of Animal Industry recommends the following mixture as a cheap and effective compound for destroying the growth of horns in calves under one month of age: 50 per cent. of caustic soda, 25 per cent. of kerosene, and 25 per cent. of water. An emulsion of the kerosene and soda is prepared by heating and vigorously stirring. This is then dissolved in water. Care should be exercised that it does not come in contact with anything but the horny protuberance.

London, England, reported another outbreak of contagious pleuro-pneumonia in April, appearing in the district of Whitechapel.

*Modern Medicine and Bacteriological Review*, in the June number, says: "From an extended series of thoroughly scientific experiments we learn that milk from cows with tuberculous udders is of an extremely virulent nature; further, buttermilk, cream, and butter derived from such milk contain tuberculous matter actively injurious to man. Since we can, as a rule, know nothing of the condition of the cows from which we obtain our milk, it must be evident that all dairy-milk should be boiled before being placed upon our table."

Chief Milk-inspector Byrnes, of Philadelphia, finds the pure-food act passed by the last State Legislature a very efficient aid in dealing with those who violate the laws in offering for sale impure milk or milk-products not up to the standard.

Infectious pneumonia among horses has been quite prevalent the past three months among many of the large stables in several of the large Eastern cities. The fatalities have been quite numerous.

Equal parts of acetanilid and boric acid as a dressing-powder, after cleansing the parts with creolin, proved a very satisfactory and prompt healing medium in the after-treatment following the removal of a fatty tumor, weighing thirteen ounces, from the base of the ear of a highly-bred trotting-mare at the Philadelphia Veterinary Sanitarium.

A very curious case was presented at the Philadelphia Veterinary Sanitarium in July for treatment. A bay gelding about thirteen years of age, used mostly for saddle purposes, owned by the present owner about nine years, during which time he had never noticed the penis protruded from the sheath in urinating. The sheath had become so offensive as to make the use of the animal very unpleasant and annoying. Examination revealed a small atrophied penis at the posterior portion of the sheath, perfect in shape and outlines; urethral opening normal; no ulceration or tenderness; an inflammatory seborrhœa of the sheath, with intense inflammation and denudation of the lining membrane of the sheath. Warm-water injections freely charged with creolin and a dusting-powder, composed of boric acid, starch, and lycopodium, insufflated over the parts, in eight days removed all offensive odor, lessened the discharge to almost nothing, and reduced the redness and excoriation of the parts.

The *Breeders' Gazette* says in regard to the horn-fly that is producing so much loss among those who give attention to the summer feeding of cattle: "Various means of protection have been offered, but most of them require so frequent renewal of the applications that the time and difficulty in so doing make them of little practical value. Prof. Scovill, of the Kentucky Experiment Station, has successfully used a kerosene emulsion applied by a small spray-pump. In a small herd this emulsion is made in the following proportions: One pint of soft soap (or one-fourth pound of hard soap dissolved in boiling water), and one pint of kerosene in fifteen pints of water, thoroughly whipped and churned together. At the Indiana Experiment Station one quart of fish-oil, to which is added two tablespoonfuls of crude carbolic acid. These liquids are applied over the body with flat paint-brushes four inches wide. The most promising plan seems to be that of breaking up the nests. The manure gathered up or broken to pieces within a day or so throughout the pastures, as in these deposits are to be found the eggs, will greatly aid their effective extermination. Dry hot weather retards their multiplication; wet weather encourages."

Dr. Cecil French reports a case of canine cryptorchid noted in a post-mortem recently held. Dr. French also reports a case of tuberculosis in a setter bitch, with nearly every organ in the body affected. Diagnosis confirmed by bacteriological investigation at the Army Medical Museum. Tuberculin injected in this animal gave no reaction.

Dr. M. Francis, of the Texas Experiment Station, reports that the use of crude cotton-seed oil in the dipping-vats, covering the water in the vats to the depth of three-fourths to one inch, and having the cattle swim through, proved a very effectual and harmless means of dealing with the ticks. Other oils are being tested.

